# Integrative multi-omics summary-based mendelian randomization identifies key oxidative stress-related genes as therapeutic targets for atrial fibrillation and flutter

**DOI:** 10.3389/fgene.2024.1447872

**Published:** 2024-09-18

**Authors:** Shijian Chen, Junlong Sun, Wen Wen, Zhenfeng Chen, Ziheng Yu

**Affiliations:** ^1^ Huzhou Central Hospital, The Fifth School of Clinical Medicine of Zhejiang Chinese Medical University, Huzhou, China; ^2^ Huzhou Central Hospital, The Affiliated Central Hospital of Huzhou University, Huzhou, China

**Keywords:** atrial fibrillation, oxidative stress, summary-based mendelian randomization, QTL, TTN

## Abstract

**Background:**

Atrial fibrillation (AF) is a prevalent cardiac arrhythmia associated with substantial morbidity and mortality. Oxidative stress (OS) has been implicated in the pathogenesis of AF, suggesting that targeting OS-related genes could offer novel therapeutic opportunities. This study aimed to identify causal OS-related genes contributing to AF through a comprehensive multi-omics Summary-based Mendelian Randomization (SMR) approach.

**Methods:**

This study integrated data from genome-wide association studies (GWAS) with methylation quantitative trait loci (mQTL), expression QTL (eQTL), and protein QTL (pQTL) to explore the relationships between oxidative stress-related (OS-related) genes and AF risk. Genes associated with oxidative stress and AF were obtained from the Nielsen et al. study (discovery) and the FinnGen study (replication). The SMR analysis and HEIDI test were utilized to assess causal associations, followed by Bayesian co-localization analysis (PPH4 > 0.5) to confirm shared causal variants. Multi-omics data were employed to analyze the associations within mQTL-eQTL pathways. A two-sample MR analysis was conducted for sensitivity verification. The significance of findings was determined using a false discovery rate (FDR) < 0.05 and *p*_HEIDI > 0.01.

**Results:**

At the DNA methylation level, 19 CpG sites near 7 unique genes were found to have causal effects on AF and strong co-localization evidence support (PPH4 > 0.70). At the gene expression level, six oxidative stress-related genes from eQTLGen and three from GTEx (v8), including *TNFSF10*, *CDKN1A*, *ALOX15*, *TTN*, *PTK2*, *ALB*, *KCNJ5*, and *CASQ2*, were found to have causal effects on AF in the sensitivity and co-localization analyses (PPH4 > 0.50). At the circulating protein level, both *ALAD* (OR 0.898, 95% CI 0.845–0.954, PPH4 = 0.67) and *APOH* (OR 0.896, 95% CI 0.844–0.952, PPH4 = 0.93) were associated with a lower risk of AF, and *APOH* was validated in the replication group. After integrating the multi-omics data between mQTL and eQTL, we identified two oxidative stress-related genes, *TTN* and *CASQ2*. The methylation of cg09915519 and cg10087519 in TTN was associated with higher expression of *TTN* and a lower risk of AF, which aligns with the negative effect of *TTN* gene expression on AF risk. *TTN* may play a protective role in AF.

**Conclusion:**

This study identified several OS-related genes, particularly TTN, as having causal roles in AF, which were verified across three-omics pathways. The findings underscore the importance of these genes in AF pathogenesis and highlight their potential as therapeutic targets. The integration of multi-omics data provides a comprehensive understanding of the molecular mechanisms underlying AF, paving the way for targeted therapeutic strategies.

## Introduction

Atrial fibrillation is the most common sustained cardiac arrhythmia encountered in clinical practice, significantly contributing to morbidity and mortality globally (Sagris et al., 2021). The pathogenesis of AF is complex, involving various factors such as electrical and mechanical changes in the atria, inflammation, oxidative stress, and fibrosis (Gaudino et al., 2023). Inflammation has been well-documented as a critical factor in the development and progression of AF, leading to atrial fibrosis and structural remodeling. Similarly, OS, characterized by the excessive production of reactive oxygen species (ROS), has been implicated in atrial remodeling and the onset of AF (Ramos-Mondragón et al., 2023).

Previous studies have highlighted the interplay between inflammation and OS in the pathophysiology of AF (Ramos-Mondragón et al., 2023; Balan et al., 2024). Inflammatory markers such as high-sensitivity C-reactive protein and galectin-3 have been associated with AF incidence and recurrence, suggesting their role in atrial fibrosis and remodeling (Noubiap et al., 2021). Additionally, OS markers like myeloperoxidase and oxidized low-density lipoprotein have been linked to cardiac tissue damage and AF (Rafaqat et al., 2021). Despite these associations, the relative contributions of inflammation and OS to AF and their potential interactions remain not fully understood.

Recent advancements in multi-omics technologies provide a unique opportunity to investigate the molecular mechanisms underlying AF by integrating genomic, epigenomic, and proteomic data. Summary-based Mendelian randomization is a powerful analytical approach that leverages genetic variants as instrumental variables to infer causal relationships between risk factors and disease outcomes. By combining SMR with multi-omics data, it is possible to identify causal genes and pathways involved in AF, offering insights into potential therapeutic targets.

In this study, we aimed to identify therapeutic genes for AF by GWAS with mQTL, eQTL, and pQTL data using a comprehensive SMR approach. We hypothesized that OS-related genes play a causal role in AF and that their identification could provide novel targets for therapeutic intervention. Through rigorous statistical analysis and integration of multi-omics data, we sought to elucidate the molecular mechanisms linking OS to AF and highlight key genes for further investigation and potential therapeutic development.

## Methods

### Study design

**FIGURE 1:**
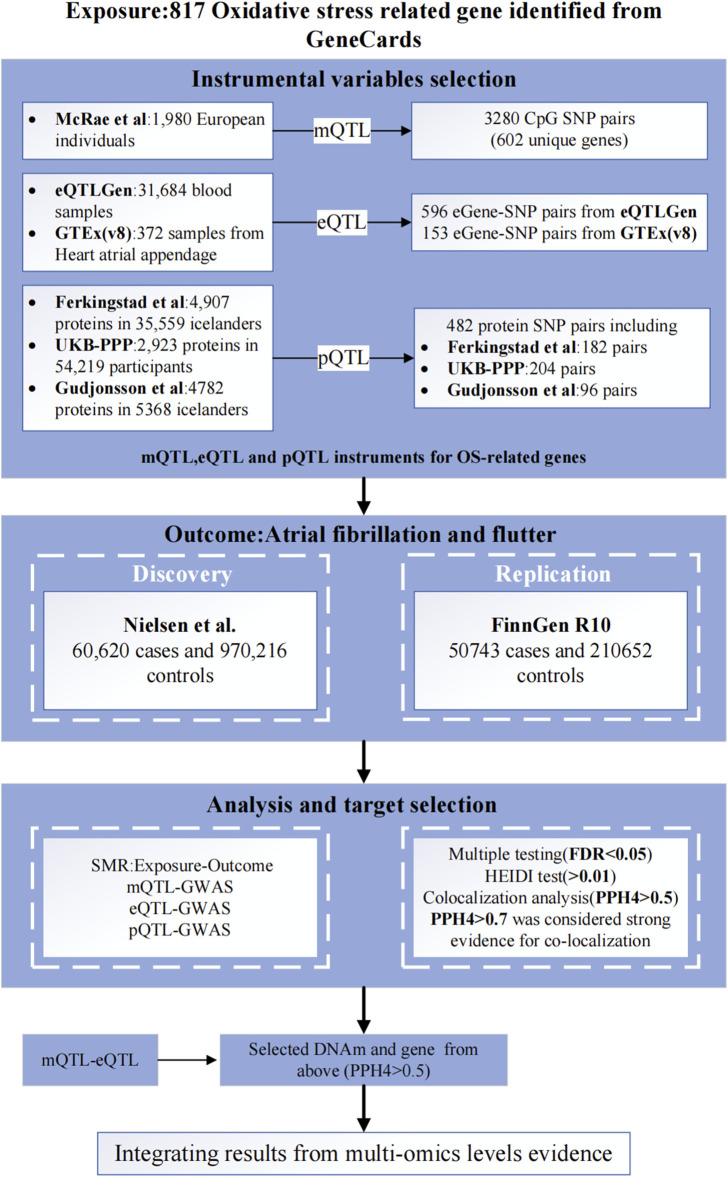
Study design. SMR, summary-based Mendelian randomization; QTL, quantitative trait loci; SNP, single nucleotide polymorphisms; OS, oxidative stress; GWAS, Genome-Wide Association Study; FDR, False Discovery Rate; PPH4, posterior probability of H4.

Figure 1 illustrates the comprehensive design of the study. Initially, we identified 817 OS-related genes from the GeneCards database. Next, we integrated GWAS of AF with blood expression QTL (eQTL), methylation QTL (mQTL), and protein QTL (pQTL) summary data for analysis using the SMR method. Then, we identified potential causal OS-related genes through SMR analysis and Bayesian co-localization analysis, we also analyzed the molecular networks of OS-related genes within mQTL and eQTL through SMR analysis. Finally, we validated the robustness of the primary findings using a two-sample MR analysis (Supplementary Table S17).

### Data sources

The GWAS by Nielsen et al. encompassed 1,030,836 participants (60,620 AF cases and 970,216 controls) of European descent (Nielsen et al., 2018). The median age was not provided, and 53% of the participants were women. Summary-level data of genetic associations with AF were also obtained from the publicly available R10 data release of the FinnGen study (Kurki et al., 2023). The diagnosis of AF was based on ICD codes and confirmed by Social Insurance Institution codes, with a total of 50,743 AF cases and 210,652 controls. The discovery stage of the research utilized the Nielsen et al. study, while the replication stage used data from the FinnGen study.

By integrating multi-omic data, it is possible to illuminate the molecular networks underlying mitochondrial dysfunction. Quantitative trait loci (QTL) studies facilitate the understanding of associations between single nucleotide polymorphisms (SNPs) and levels of DNA methylation, gene expression, and protein abundance.

McRae et al. reported data on SNP-CpG associations in blood from a study of 1,980 individuals of European descent, focusing on mQTLs (McRae et al., 2018). These data were normalized using a generalized linear model that accounted for factors such as chip type, sex, age, age squared, and their interactions (McRae et al., 2018).

Gene expression data were sourced from the eQTLGen consortium, which offered a substantial sample size (n = 31,684) to identify SNPs associated with the expression of genes targeted by the corresponding plasma proteins (Võsa et al., 2021).

For protein abundance, summary statistics were obtained from three independent pQTL studies conducted by Ferkingstad et al., Gudjonsson et al., and the UKB-PPP. For Ferkingstad et al., 28,191 genetic associations (*p* < 1.8 × 10^−9^) for 4,907 aptamers were identified in 35,559 Icelanders based on the SomaScan platform (Ferkingstad et al., 2021). For Gudjonsson et al., 7,506,463 genetic associations (*p* < 1.046 × 10^−11^) for 4,782 serum proteins encoded by 4,135 unique human genes in the population-based AGES cohort of 5,368 elderly Icelanders were measured by the slow-off rate modified aptamer (SOMAmer) platform (Gudjonsson et al., 2022). For the UKB-PPP, a total of 23,588 primary (sentinel) genetic associations (*p* < 1.7 × 10^−11^, clumping ±1 Mb, r^2^ < 0.8) for 2,923 proteins in 54,219 participants from the UK Biobank Pharma Proteomics Project (UKB-PPP) were identified using the Olink platform (Sun et al., 2023). The protein levels were adjusted using rank-inverse normal transformation, taking into account age, sex, and sample storage age.

The tissue-specific expression of target genes that could potentially cause AF was further assessed using tissue-specific eQTL data from the Genotype Tissue Expression (GTEx) web portal (https://gtexportal.org/home/). The GTEx dataset includes information from 838 donors and 17,382 samples spanning 52 tissues and 2 cell lines (GTEx Consortium., 2020). This extensive resource enabled the exploration of gene expression across a variety of tissues, providing insights into the role of specific genes in AF pathogenesis.

OS-related genes were identified from the GeneCards database (version 5.10, https://www.genecards.org) using the keyword “oxidative stress” and a relevance score of 7 or higher, following previously established methods (Qiu et al., 2020; Fan et al., 2022; Sun et al., 2022). Ultimately, we identified 817 OS-related genes (Supplementary Table S1).

After the filtering process for OS-related genes, we identified 602 genes associated with methylation, 596 with expression from eQTLGen, 153 with expression from GTEx (v8), and 482 proteins with available instruments (mQTLs, eQTLs, and pQTLs with P < 5 × 10^−8^) from the respective datasets.

### Summary-data-based MR analysis

SMR analysis was employed to assess the association between OS-related gene methylation, expression, and protein abundance with the risk of atrial fibrillation and flutter (Zhu et al., 2016). We constructed a hypothetical model of the mediation mechanism in which a single SNP influences a trait by altering the DNA methylation (DNAm) level, which in turn regulates the expression levels of a functional gene. Therefore, we also used the SMR to analyze the OS-related DNAm and gene within the multi-omics respectively (mQTL-eQTL). Utilizing the top associated cis-QTLs, the SMR method achieved significantly greater statistical power compared to conventional MR analysis, particularly when exposure and outcome data are derived from two independent samples with large sample sizes. The top associated cis-QTLs were selected by considering a window centered around each relevant gene (±1,000 kb) and meeting a *p*-value threshold of 5.0 × 10^−8^. SNPs with allele frequency differences exceeding the specified threshold (0.2 in this study) between any pairwise datasets, including the LD reference sample, the QTL summary data, and the outcome summary data, were excluded. The heterogeneity in the dependent instrument (HEIDI) test was employed to distinguish between pleiotropy and linkage, with P_HEIDI < 0.01 indicating likely pleiotropy, leading to exclusion from the analysis. P-values were adjusted to control the false discovery rate (FDR) at α = 0.05 using the Benjamini–Hochberg method. Associations with FDR-corrected P-values <0.05 and P_HEIDI >0.01 were subjected to co-localization analysis.

The SMR analysis and HEIDI test were performed using version 1.3.1 of the SMR software (https://yanglab.westlake.edu.cn/software/smr/#Download). Two-sample MR analysis were conducted using the “TwoSampleMR (version 0.5.6)” package of the R software (version 4.2.2).

### Co-localization analysis

We conducted co-localization analyses to identify shared causal variants between AF and identified OS-related mQTLs, eQTLs, or pQTLs using the coloc R package (version 5.2.2) (Giambartolomei et al., 2014). In these analyses, five distinct posterior probabilities are reported, corresponding to the following five hypotheses: 1) no causal variants for either of the two traits (H0); 2) a causal variant for gene expression only (H1); 3) a causal variant for disease risk only (H2); 4) distinct causal variants for each trait (H3); and 5) a shared causal variant for both traits (H4). For the co-localization of pQTL-GWAS, eQTL-GWAS, and mQTL-GWAS, the colocalization region windows were all set at ±1,000 kb, ±1,000 kb, ±500 kb, respectively. The prior probabilities that the causal variants are associated with only trait 1, only trait 2 (AF), and both are respectively set at 1.0 × 10^−4^, 1.0 × 10^−4^, and 1.0 × 10^−5^. A posterior probability of H4 (PPH4) > 0.5 was considered evidence of co-localization, with this threshold corresponding to a false discovery rate of P-values <0.05, thereby reinforcing the evidence for a causal relationship (Huang et al., 2023).

### Integrating results from multi-omics level of evidence

To obtain a full picture of the associations between the regulation of OS-related genes and AF at the genomic level, we conducted SMR analysis of the causal associations within OS-related gene methylation and expression, in order to explore the basic mechanism (FDR < 0.05, P_HEIDI > 0.05). We focused only on the genes that passed the screening criteria. The identification of the final putative causal relationships was defined as: 1) false discovery rate (FDR) < 0.05 in all three-step SMR; 2) P_HEIDI > 0.01 in mQTL-GWAS and eQTL-GWAS SMR, P_HEIDI > 0.05 in mQTL-eQTL SMR; 3) PPH4 > 0.5 in co-localization analysis of eQTL and AF GWAS; 4) the eQTL and mQTL should correspond to the same gene symbol.

## Results

### Oxidative stress-related gene methylation and AF GWAS data

Results for causal effects of oxidative stress-related gene methylation on AF are visualized in (Table 1). After the removal of associations with P HEIDI <0.01, a total of 346 CpG sites near 159 unique genes passed the marginal significance (P_SMR_ < 0.05) (Supplementary Table S2). After correction for multiple testing (FDR < 0.05), we identified 40 CpG sites near 17 unique genes (Supplementary Table S3). 19 near 7 unique genes were found to have strong co-localization evidence support (PPH4 > 0.70) including *MAPT* (cg02228913, cg21705961, cg05301556, cg23202277, cg05772917, cg07163735, cg01934064, cg00846647), *CRHR1* (cg16228356, cg23762722, cg05727186, cg24063856, cg16642545), *TTN* (cg09915519, cg10087519), *ALPP* (cg14659346), *CREBBP* (cg05194552), *GPX4* (cg04903600) and *MAP2K2* (cg21124940) (Table 1).

**TABLE 1:** Oxidative stress gene methylation and AF GWAS data.

Gene	ProbeID	TopSNP	β coefficient	Or (95% CI)	FDR	*p* HEIDI	PPH4
*MAPT*	cg02228913	rs112572874	−0.033	0.967 (0.955.0.979)	2.27E-05	0.01	0.77
	cg21705961	rs17689,882	−0.063	0.939 (0.917.0.961)	3.52E-05	0.10	0.82
	cg05301556	rs10445363	−0.081	0.923 (0.894.0.952)	1.35E-04	0.14	0.83
	cg23202277	rs112995313	0.117	1.124 (1.073.1.178)	1.59E-04	0.23	0.83
	cg05772917	rs112155389	0.156	1.169 (1.092.1.251)	9.97E-04	0.04	0.83
	cg07163735	rs538628	0.173	1.189 (1.101.1.284)	1.29E-03	0.10	0.80
	cg01934064	rs2668668	0.089	1.093 (1.042.1.145)	1.67E-02	0.14	0.79
	cg00846647	rs111751251	−0.038	0.963 (0.943.0.983)	2.16E-02	0.09	0.76
*CRHR1*	cg16228356	rs17689,882	−0.051	0.951 (0.933.0.968)	2.92E-05	0.05	0.83
	cg23762722	rs112746008	−0.078	0.925 (0.898.0.953)	4.34E-05	0.09	0.78
	cg05727186	rs62056790	−0.095	0.91 (0.877.0.944)	1.14E-04	0.12	0.81
	cg24063856	rs62057073	−0.140	0.87 (0.817.0.926)	1.63E-03	0.08	0.73
	cg16642545	rs111273167	−0.172	0.842 (0.769.0.923)	1.67E-02	0.03	0.77
*TTN*	cg09915519	rs744426	−0.256	0.774 (0.713.0.84)	6.07E-07	0.27	0.93
	cg10087519	rs12998857	−0.364	0.695 (0.597.0.808)	3.65E-04	0.29	0.93
*ALPP*	cg14659346	rs1048995	−0.037	0.964 (0.945.0.984)	2.29E-02	0.61	0.80
*CREBBP*	cg05194552	rs2530890	−0.149	0.862 (0.794.0.935)	2.29E-02	0.26	0.96
*GPX4*	cg04903600	rs62131210	0.095	1.099 (1.045.1.157)	1.67E-02	0.05	0.83
*MAP2K2*	cg21124940	rs60505738	−0.037	0.963 (0.944.0.983)	1.68E-02	0.95	0.85

SNP, single nucleotide polymorphisms; OR, odds ratio; CI, confidence interval; FDR, false discovery rate; PPH4, posterior probability of H4.

The direction of effect estimates were not always consistent for different CpG sites located in the same gene. For example, one SD increase in genetically predicted *MAPT* methylation at cg02228913 was associated with a decreased risk of AF (OR 0.967, 95% confidence interval [CI] 0.955–0.979), whereas one SD increase in genetically predicted *MAPT* methylation at cg07163735 was associated with a higher risk of AF (OR 1.189, 95% CI 1.101–1.284). Among these identified CpG sites, the associations for cg09915519 and cg10087519 near *TTN* were replicated in FinnGen with strong co-localization evidence support (PPH4 >0.70) (Supplementary Table S4).

### Oxidative stress-related gene expression and AF GWAS data

Results for causal effects of oxidative stress related gene expression on AF are presented in (Table 2). In total, 83 associations were identified to be associated with AF at the nominally significant level (P_SMR_ < 0.05, P HEIDI > 0.01) (Supplementary Tab le S5). After multiple testing correction and co-localization analysis (Supplementary Table S6), we selected those genes which PPH4 > 0.5. Genetically predicted higher levels expression of *TNFSF10* (OR 1.160, 95% CI 1.074–1.254; PPH4 = 0.65), *CDKN1A* (OR 1.214, 95% CI 1.125–1.309; PPH4 = 0.64) and *ALOX15*(OR 1.063, 95% CI 1.028–1.099; PPH4 = 0.60) were positively associated with AF risk. Conversely, genetically predicted higher levels expression of *TTN* (OR 0.291, 95% CI 0.186–0.454; PPH4 = 0.65), *PTK2* (OR 0.794, 95% CI 0.730–0.864; PPH4 = 0.67) and *ALB*(OR 0.291, 95% CI 0.186–0.454; PPH4 = 0.98) were inversely associated with AF risk (Table 2). Associations for *TTN* and *PTK2* were all replicated in FinnGen, of which the genetically predicted high level expressions were negatively associated with AF risk (OR 0.181, 95% CI 0.098–0.336; OR 0.744, 95% CI 0.661–0.837, respectively). whereas *CDKN1A* replicated in FinnGen was positively associated with AF risk (OR 1.36, 95% CI 1.21–1.529, Supplementary Table S7).

**TABLE 2:** Oxidative stress gene expression and AF GWAS data.

Gene	TopSNP	β coefficient	Or (95% CI)	FDR	*p* HEIDI	PPH4
*TTN*	rs13034990	−1.235	0.291 (0.186.0.454)	1.48E-05	0.04	0.65
*PTK2*	rs10088133	−0.230	0.794 (0.73.0.864)	1.48E-05	0.41	0.67
*CDKN1A*	rs12199346	0.194	1.214 (1.125.1.309)	5.17E-05	0.02	0.64
*ALB*	rs150643605	−0.421	0.656 (0.529.0.815)	7.89E-03	0.04	0.98
*TNFSF10*	rs3181140	0.149	1.16 (1.074.1.254)	9.64E-03	0.59	0.65
*ALOX15*	rs72835630	0.061	1.063 (1.028.1.099)	1.54E-02	0.47	0.60

SNP, single nucleotide polymorphisms; OR, odds ratio; CI, confidence interval; FDR, false discovery rate; PPH4, posterior probability of H4.

### Tissue-specific validation

Causal associations of the expression of identified genes with AF were further explored in atrial tissue (Table 3). In total, 22associations were identified to be associated with AF at the nominally significant level (P_SMR_ < 0.05, P HEIDI > 0.01) (Supplementary Table S8). After multiple testing correction and co-localization analysis (Supplementary Table S9), genetically predicted expression levels of *KCNJ5* was associated with increases in AF risk in atrial appendage (OR 1.293, 95% CI 1.169–1.431; PPH4 = 0.97). In contrast, genetically predicted expression levels of *CASQ2* and *ALOX15* were associated with reduction in AF risk in atrial appendage tissue (OR 0.652, 95% CI 0.538–0.789; PPH4 = 0.98 and OR 0.915, 95% CI 0.867–0.966; PPH4 = 0.76, respectively). *KCNJ5* was replicated in the FinnGen study (Supplementary Table S10).

**TABLE 3:** Tissue-specific oxidative stress-related gene expression and AF GWAS data.

Gene	Tissue	TopSNP	β coefficient	Or (95% CI)	FDR	*p* HEIDI	PPH4
*KCNJ5*	Atrial appendage	rs78907918	0.257	1.293 (1.169.1.431)	9.85E-05	0.06	0.97
*CASQ2*	Atrial appendage	rs4073778	−0.428	0.652 (0.538.0.789)	7.18E-04	0.64	0.98
*ALOX15*	Atrial appendage	rs8071990	−0.089	0.915 (0.867.0.966)	3.62E-02	0.54	0.76

SNP, single nucleotide polymorphisms; OR, odds ratio; CI, confidence interval; FDR, false discovery rate; PPH4, posterior probability of H4.

### Oxidative stress-related gene relative proteins and AF GWAS data

There were 36 oxidative stress related proteins from 3 independent researches including Ferkingstad study, Gudjonsson study and UKB-PPP Olink which separately associated with AF risk at P_SMR_ < 0.05 level (Supplementary Table S11). After adjustment for multiple testing, we obtained 2 proteins including *ALAD*, *APOH* (Supplementary Table S12). Both *ALAD* (Ferkingstad study) and *APOH*(Gudjonsson study) were associated with a lower risk of AF(OR 0.898, 95% CI 0.845–0.954, PPH4 = 0.67; OR 0.896, 95% CI 0.844–0.952, PPH4 = 0.93, respectively). *APOH* was replicated in the FinnGen study (Supplementary Table S13).

### SMR analysis for mQTL and eQTL data

**FIGURE 2:**
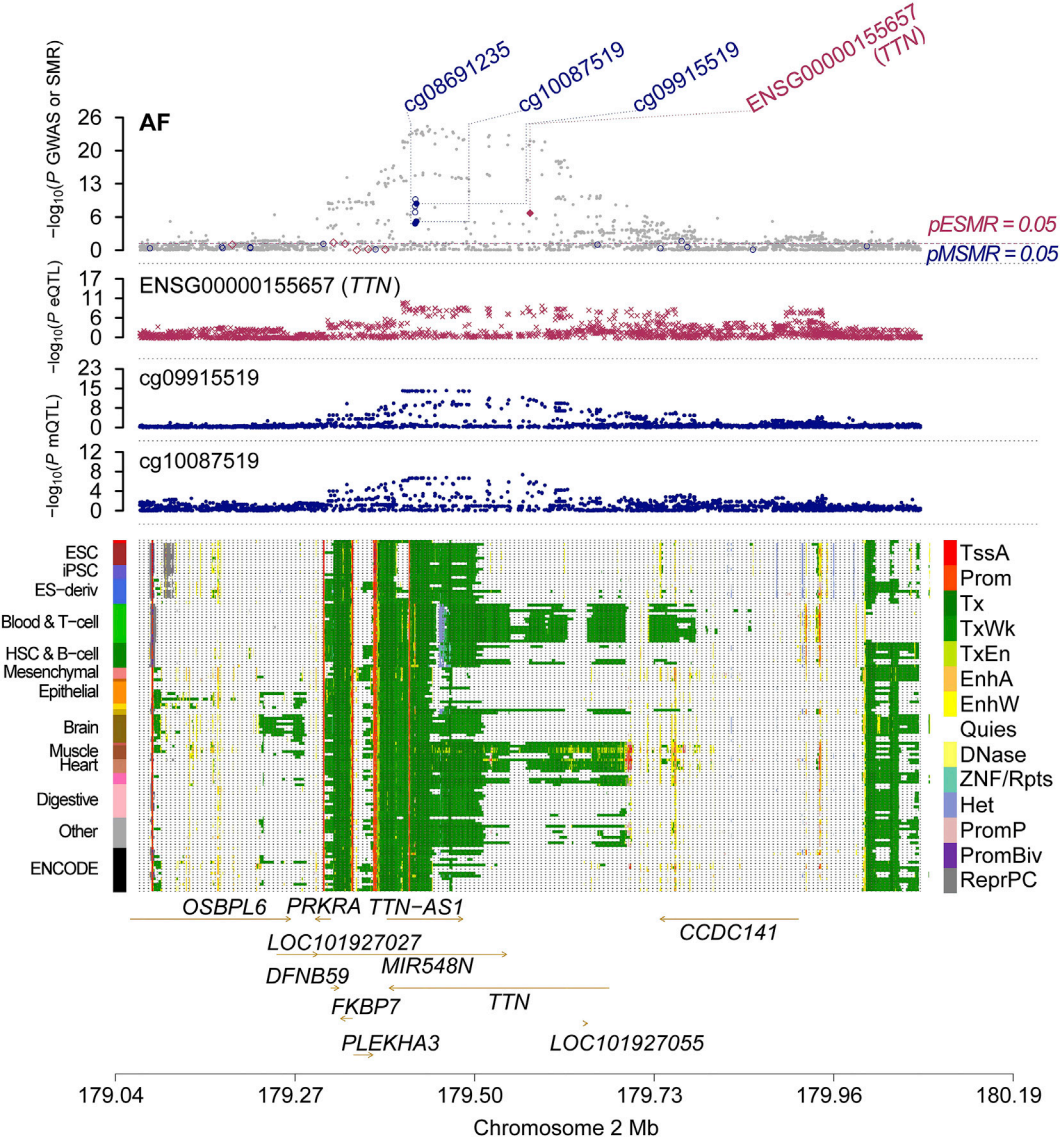
Multi-omics data integration for AF. Results of associations across mQTL, eQTL and GWAS at the TTN locus.

**FIGURE 3:**
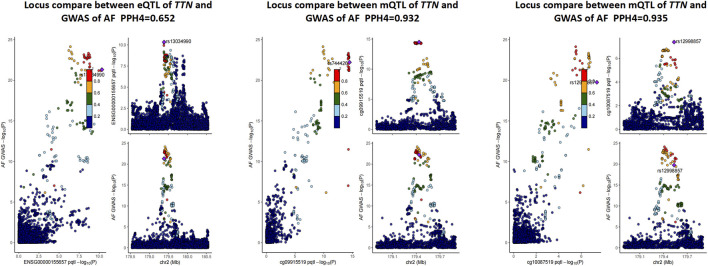
Result of co-localization analysis in mQTL, eQTL of *TTN* and GWAS of AF. QTL, quantitative trait loci; GWAS, Genome-Wide Association Study; PPH4, posterior probability of H4.

It is well known that gene methylation affects gene expression. Therefore, we proceeded to explore the possible link between DNAm and gene expression by using DNAm as the exposure and transcripts as the outcome. After screening the results by FDR < 0.05 and P HEID > 0.05, we obtained the regulatory relationships for the expression of 252 OS-related genes regulated by 595 DNA methylation CpG sites (Supplementary Table S14). We only analyse the genes which have higher co-localization evidence (PPH4 > 0.5) support with AF (Table 2). Finally we identified 1 OS-related genes regulated by 2 DNA methylation CpG sites (Supplementary Table S16; Figure 2). *TTN*, there were two significantly associated methylation sites (cg09915519 and cg10087519), two of which were positively correlated with *TTN* expression and passed the co-localization analysis in mQTL-GWAS (Figure 3).

The top plot shows −log10 (p-values) of SNP from GWAS. The red diamonds and blue circles represent −log10 (p-values) from SMR tests for associations of gene expression and DNAm probes with trait, respectively. The solid diamonds and circles are the probes not rejected by the HEIDI test. The second plot shows −log10 (p-values) of the SNP associations for gene expression probe ENSG00000155657 (*TTN*). The third plot shows −log10 (p-values) of the SNP associations for DNAm probes. The bottom plot shows 14 chromatin state annotations (indicated by colours) of 127 samples from the REMC for different primary cells and tissue types (rows).

### SMR analysis for mQTL and eQTL from GTEx(v8) tissue data

After screening the results by FDR < 0.05 and P HEID > 0.05, we obtained the regulatory relationships for the expression of 92 OS-related genes from tissue of heart atrial appendage regulated by 256 DNA methylation CpG sites (Supplementary Table S13). We analyse the genes which have co-localization evidence (PPH4>0.5) support with AF (Table 2). Finally we identified *CASQ2* negatively regulated by cg20810993 CpG sites.

### Multi-omics level integration

After integrating evidence from multi-omics data, we identified *TTN* from eQTLGen and *CASQ2* from GTEx (v8) with higher evidence linking them to AF through the mQTL to eQTL pathway. We constructed a hypothetical model of the mediation mechanism: a single nucleotide polymorphism (SNP) affects the trait by altering DNA methylation (DNAm) levels, which then regulate the expression of a functional gene. One CpG sites (cg20810993) were excluded because it did not show significant results in the mQTL-GWAS SMR analysis.

**FIGURE 4:**
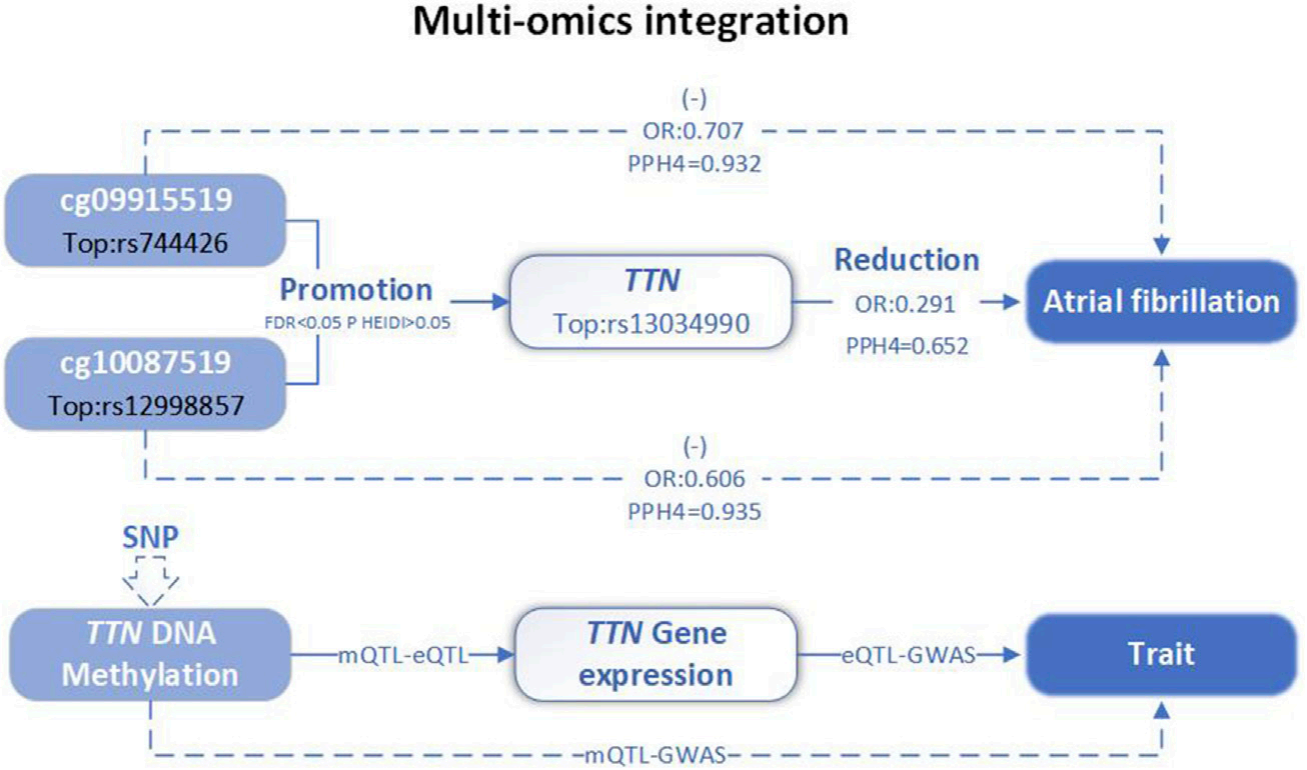
Multi-omics level integration of *TTN*. SNP, single nucleotide polymorphisms; QTL, quantitative trait loci; Top, top SNP; FDR, false discovery rate; PPH4, posterior probability of H4; OR, odds ratio.

In conclusion, the integration analysis results demonstrated that *TTN* methylation regulated by cg09915519 and cg10087519 increases *TTN* expression, which is associated with a lower risk of AF (Figure 4).

## Discussion

In this study, we employed an integrative multi-omics summary-based Mendelian randomization and Bayesian co-localization to identify key oxidative stress-related genes as therapeutic targets for AF. Integration of AF GWAS summary data and mQTL and eQTL for OS-related genes prioritized one gene expression (*TTN*) and two CpG sites (cg09915519 and cg10087519). Our findings significantly adance the understanding of the genetic underpinnings of AF, particularly in the context of oxidative stress pathways.

*TTN* (Titin) is an essential structural and functional protein in muscle tissue, particularly within the sarcomeres of cardiac muscle (Loescher et al., 2022). Mutations in the *TTN* gene, particularly truncating variants (TTNtv), are associated with various cardiomyopathies, most notably dilated cardiomyopathy (Santiago et al., 2021). Research by Akhtar et al. involved 537 individuals from 14 centers, with a median follow-up of 49 months, and concluded that TTNtv is associated with frequent arrhythmias and heart failure, with male sex and left ventricular systolic dysfunction being significant predictors of poor outcomes (Akhtar et al., 2020). TTNtv can lead to sarcomere insufficiency and altered mechanical properties of cardiac muscle (Fomin et al., 2021). These mutations can disrupt the ubiquitin-proteasome system and autophagy pathways, contributing to the accumulation of defective proteins and increased cellular stress (Müller et al., 2021). TTNtv are significantly associated with early-onset AF, especially in patients without traditional risk factors (Andreasen et al., 2020). Yoneda et al. recruited 1,293 participants who underwent whole genome sequencing, focusing on 145 genes associated with cardiomyopathy and arrhythmia syndromes. After screening, the most frequently affected genes included *TTN*, *MYH7*, *MYH6*, *LMNA*, and *KCNQ1*, with *TTN* variants being the most prevalent (Yoneda et al., 2021). The structural abnormalities caused by TTNtv, such as atrial dilation and fibrosis, contribute to the development of AF (Rudaka et al., 2023). TTNtv can act as a monogenic cause of AF, as the European Society of Cardiology reported that *TTN* gene variants were notably enriched in early-onset lone AF cases (Lazarte et al., 2021). The study concludes that lone AF is associated with an enrichment of rare loss-of-function variants in ventricular cardiomyopathy genes, particularly in the *TTN* gene, which suggests that atrial cardiomyopathy might be a genetic sub-phenotype of AF (Lazarte et al., 2021).

The expression of the *TTN* gene is primarily regulated by several CpG sites, with cg09915519 and cg10087519 being the most significant. Grzeczka et al. found that DNA methylation, particularly of certain CpG sites, was found to significantly affect the expression of genes involved in AF. Hypermethylation of key regulatory genes in atrial tissue, such as those involved in ion channel function and fibrosis, was associated with increased risk of AF (Grzeczka et al., 2023).

Ongoing research aims to translate these genetic insights into effective therapeutic strategies. Gene-editing technologies, such as CRISPR/Cas9, hold promise for correcting *TTN* mutations, thereby restoring normal protein function and enhancing cardiac contractility (Kang et al., 2022). Our findings support the hypothesis that certain benign mutations in *TTN* may reduce the risk of AF, thereby exerting a protective effect on the pathological processes underlying AF. Additionally, our SMR analysis provided evidence that the upregulation of methylation at cg09915519 and cg10087519 is associated with a decreased risk of AF through increased *TTN* expression levels.

In the transcriptomic and multi-omics studies of atrial tissue, we found that the expression of *CASQ2* is associated with the occurrence of AF (OR 0.652, 95% CI 0.538–0.789, PPH4 = 0.98). *CASQ2* (calsequestrin 2) is a calcium-binding protein primarily located in the sarcoplasmic reticulum of cardiac muscle cells. It plays a crucial role in calcium homeostasis by regulating calcium storage and release, which is essential for proper cardiac muscle contraction (Ng et al., 2020). Abnormalities in calcium handling are known to contribute to various cardiac arrhythmias, including ventricular tachycardia (Askarinejad et al., 2024). Studies have shown that altered *CASQ2* expression may contribute to the development of AF by affecting the stability of intracellular calcium levels, leading to increased susceptibility to abnormal electrical activity in the atria (Wang et al., 2020). However, we were unable to identify the cg20810993 site from the mQTL-AF GWAS analysis in both the discovery and replication groups, which was expected to regulate the expression of *CASQ2*.

Our study involved three independent studies searching for the OS-related protein that regulated the risk of AF. *APOH* was associated with a lower risk of AF (PPH4 = 0.93), a finding that was also replicated in the FinnGen study. *APOH*, also known as apolipoprotein H or beta-2-glycoprotein I, is a multifunctional protein primarily involved in lipid metabolism and immune regulation (Kamboh and Mehdi, 1998). Hoekstra et al. identified a novel variant in the *APOH* gene through a GWAS involving 293,274 White British individuals from the United Kingdom Biobank, which was significantly associated with increased Lp(a) levels. The variant rs8178824 in *APOH* showed a strong association, even after adjusting for known variants in the LPA region (Hoekstra et al., 2021). Masson et al. conducted a systematic review to investigate the association between elevated Lp(a) levels and AF (Masson et al., 2023). Some studies found no significant association between Lp(a) levels and AF, while others reported both positive and inverse relationships. For instance, a Chinese study showed an inverse association between Lp(a) levels and AF, while other studies found no significant link or even a positive association (Volgman et al., 2024; Yang et al., 2023). Therefore, the causal relationship between *APOH* and AF remains controversial, and larger-scale studies are needed to further substantiate this.

While our multi-omics SMR approach is robust, several limitations should be noted. The population-specific nature of the datasets used, primarily of European descent, may limit the generalizability of our findings to other ethnic groups. Future research will aim to replicate our findings in more diverse populations to enhance the generalizability of the therapeutic targets identified. The current study was constrained by the limited availability of OS-related proteins within the pQTL dataset, preventing a comprehensive exploration of the causal relationship between oxidative stress proteins and AF risk. Although we conducted tissue-specific analyses of candidate genes using databases derived from the atrial appendage, the genetic data obtained from circulating blood may not fully represent the status of OS-related genes in myocardial tissue. Additionally, our analysis was restricted to cis-eQTL and cis-mQTL data for OS-related genes, leaving the potential influence of trans-eQTL and trans-mQTL data on the regulatory network largely unexplored. The cross-sectional nature of the data used limits our ability to observe the progression of AF over time, which is vital for understanding the disease’s development and the long-term effects of potential genetic risk factors. Moreover, functional validation of our findings through experimental studies remains necessary. Considering the multifactorial influences on OS gene expression, we anticipate that integrating data across multiple molecular levels with GWAS data could yield novel insights in future research.

## Conclusion

Our study identifies TTN as key OS-related genes with causal roles in AF, providing novel insights into AF pathogenesis and potential therapeutic targets. The integration of genetic, epigenetic, and proteomic data offers a powerful approach to uncovering the complex molecular underpinnings of AF, paving the way for precision medicine strategies in AF management.

## Data Availability

The original contributions presented in the study are publicly available. This data can be found here: Atrial fibrillation from Nielsen JB: https://gwas.mrcieu.ac.uk/datasets/ebi-a-GCST006414/; Atrial fibrillation from FinnGen: https://storage.googleapis.com/finngen-public-data-r10/summary_stats/finngen_R10_I9_AF.gz; Decode 2021: Large-scale integration of the plasma proteome with genetics and disease | Nature Genetics: https://www.nature.com/articles/s41588-021-00978-w; UKB-PPP: Plasma proteomic associations with genetics and health in the UK Biobank | Nature https://www.nature.com/articles/s41586-023-06592-6; Gudjonsson: A genome-wide association study of serum proteins reveals shared loci with common diseases | Nature Communications: https://www.nature.com/articles/s41467-021-27850-z; GTEx: GTEx Consortium (2020): https://gtexportal.org/home/; Data for SMR was downloaded from: https://yanglab.westlake.edu.cn/software/smr/#eQTLsummarydata; mQTL summary data: Identification of 55,000 Replicated DNA Methylation QTL | Scientific Reports (nature.com): https://www.nature.com/articles/s41598-018-35871-w; Data for SMR was downloaded from: https://yanglab.westlake.edu.cn/software/smr/#mQTLsummarydata; eQTL data Large-scale cis- and trans-eQTL analyses identify thousands of genetic loci and polygenic scores that regulate blood gene expression | Nature Genetics: https://www.nature.com/articles/s41588-021-00913-z; Data for SMR was downloaded from: https://yanglab.westlake.edu.cn/software/smr/#eQTLsummarydata.
